# Taking Justice Into Their Own Hands: Predictors of Netilantism Among Cyber Citizens in Hong Kong

**DOI:** 10.3389/fpsyg.2020.556903

**Published:** 2020-10-02

**Authors:** Lennon Y. C. Chang, Jinxin Zhu

**Affiliations:** ^1^School of Social Sciences, Monash University, Clayton, VIC, Australia; ^2^Assessment Research Centre, The Education University of Hong Kong, Tai Po, Hong Kong

**Keywords:** internet vigilantism (netilantism), confidence in criminal justice system, cyber crowdsourcing, social justice, human flesh searching

## Abstract

This research examined the characteristics and predicting indicators of netizens which contribute to “Human Flesh Searching” and internet vigilantism. Human Flesh Searching (HFS) is a form of collective online behavior where netizens contribute information to social media and/or networking platforms about a certain event or a target individual or group to achieve what they regard as justice. It has been used to identify and investigate crime. Some netizens go further and take justice into their own hands by punishing alleged criminals and deviants through online shaming. Using the results of a survey conducted in Hong Kong, the research found both gender and time spent online are not significant variables to predict netizens’ intention to contribute to HFS. A positive attitude toward HFS was the strongest predictor of HFS intention. Vigilantism was also a strong predictor of HFS intention. Vigilantism not only affects HFS intention directly, but also indirectly through a positive attitude on HFS. Fairness might negatively influence people’s HFS intention and attitude toward HFS; however, this influence was found to be weak in the present study. Social Justice might not affect HFS intention directly, yet it might exert its effect via a positive attitude toward HFS. That is, netizens who intend to contribute to HFS are those who have less confidence in the criminal justice system and believe highly that people should take justice into their own hands.

## Introduction

Technology has changed every aspect of our everyday lives. People now do a lot of things through the internet without physical contact. During the COVID-19 lockdown, we saw how people sought to maintain their normal lives without going out. People talked to each other online via social media such as Facebook, Line, WhatsApp, and WeChat. Conferencing apps such as Zoom made it possible for people to organize not only meetings but also parties online. Thanks to these conferencing applications such as Zoom and Cisco WebEx, online teaching and working from home became a “new normal” during the period of lockdown and people even organized virtual social activities such as drinks and parties using new technologies. We also see netizens using the internet, social media and online platforms to investigate crime, to report issues as online journalists and to pass judgment.

Using the skill of cyber-crowdsourcing, “netizens” (citizens actively involved in the online community) can provide information and clues about crime or deviant behavior. Fellow netizens may then conduct further investigations to dig out more information based on the initial information and clues provided. Examples can be seen in online responses which *identify* crime (for example, anti-corruption activities in China), *investigate* crime or deviant behavior (e.g., the 2013 Boston marathon bombing in the United States and police brutality cases in Hong Kong), and/or *punish* criminals through naming and public shaming (e.g., naming and shaming alleged cyberbullies and online child-predators). As Chang and Grabosky(2017: 545) argued, cyber crowdsourcing “has been shown to be a formidable form of private regulation.”

Human Flesh Searching (HFS), known as “renrou sousou,” or “qi-di” in Chinese, is a good example of how technology is being used to achieve “justice” as perceived by netizens. HFS is a collective online behavior where netizens contribute knowledge and information through social media or networking platforms to expose alleged facts related to certain events and/or to publish information on a target individual or group. It emerged first in China in early 2000 and has become common in the Greater China Region, i.e., the People’s Republic of China (China), Hong Kong and Taiwan. Since 2010, it has become common throughout the world (Chang and Poon, 2017). While some HFS is undertaken just for fun or to fulfill one’s curiosity (such as gossip about a celebrity), most HFS is undertaken with the aim of exposing crime and deviant behavior, and to shame and punish alleged criminals and deviant individuals (Ong, 2012; Hatton, 2014; Chang and Leung, 2015). Chang and Poon (2017) coined the term “netilantism” (internet vigilantism) to describe the latter behavior.

According to Chang and Poon (2017), netilantism included behaviors such as (1) online activities to identify/disclose crime (such as identifying corrupt officials in China); (2) to investigate crime or deviant behavior (such as netizens trying to disclose the identity of police involved in violent behavior during the 2019 Anti-extradition protests in Hong Kong or 2014 Sunflower Movement in Taiwan); and (3) to punish criminals or deviants through public shaming and naming (such as public shaming of alleged child predators). Social media and networking platforms such as Facebook, Youtube, Weibo, and Telegram are used by internet vigilantes (netilantes) to post information and conduct cyber-crowdsourcing. Traditional police-initiated requests for information from the public (such as America’s Most Wanted, Crime Stoppers and *ad hoc* requests) about, for example, the identity of individuals captured on CCTV imagery, do not disclose what information the police have already gathered. The information provided by police is controlled and the information provided to them is not publicly shared. Netilantism differs from this. It provides peer-to-peer, multi-directional information sharing that can be aggregated. We also see that technology and networking platforms are being used increasingly for “sousveillance” in which netizens record and share alleged misbehavior by authorities (Mann, 2004). Although netilantism can contribute to co-production of security and cyber security, it is important to address and mitigate the risks that come with it such as the legitimacy of the information provided, the provision of false or misleading information intended to interfere with or mislead the crime investigation and the consequences that might be caused by identifying the wrong suspect (Chang, 2018; Chang et al., 2018).

Most research on HFS has been focused on HFS in China and has been published in Chinese (Li, 2008; Wang, 2009; Zhu and Liu, 2009). There has also been research on internet vigilantism that categorizes the motives of netilantes (Herold, 2011). Nhan et al. (2017), using the 2013 Boston marathon bombing as a case study, analyzed how cyber-crowdsourcing contributed to the investigation of the event and argued more research needs to be done on the forms and interaction between the police and the public. Recently, a systematic review of HFS cases in the greater China region was conducted by academics in Hong Kong (Chang and Leung, 2015; Chia, 2019a). Chang and Leung (2015) identified differences in types of HFS in Hong Kong, Taiwan, and China. Chia (2019a), using similar methods, reviewed cases in the same region in 2006–2015, through the lens of media studies. Trottier (2017, 2019) argued that weaponized visibility has become a norm in our digital era and proposed a conceptual model of digital vigilantism.

Nonetheless, despite the discussion on the impact of HFS on society and how netizens use HFS to realize their so-called “justice,” there are only a few empirical studies examining why netizens contribute to HFS. Skoric et al. (2010), using an online survey with Singaporeans, investigated the relationships between personal characteristics (extroversion, neuroticism, agreeableness, conscientiousness, and openness), Asian values and the contribution to online shaming. Chang and Poon (2017), using empowerment theory, tested the differences between netilantes, bystanders, and victims. Chia (2019b) examined the relationship between media coverage and netilantism and found favorable media coverage is essential to netilantism.

There is still little understanding of why people contribute to netilantism. Do netilantes have similar personal characteristics as vigilantes? Are they engaging in HFS to offset the inadequacy of the formal justice system? Do they have confidence in the current criminal justice system and social justice?

This research will contribute to our knowledge of netilantism from a criminological lens, seeking to understand the relationship between netizens’ attitudes toward social justice, fairness and criminal justice systems, and their intention to become netilantes.

**FIGURE 1:**
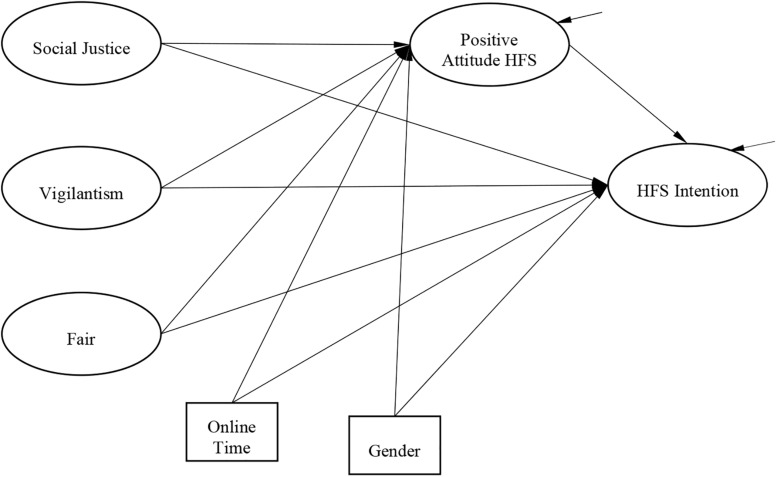
Hypothesized model. Note: Ellipses stand for latent variables and rectangles stand for observed variables.

The Theory of Planned Behavior (TPB) was developed to predict people’s intention to engage in certain behavior. As suggested by the TPB, behavioral intention can be predicted by perceived control, that is, “a person’s perception of control over behavioral performance” (Montaño and Kasprzyk, 1997: 71). Montaño and Kasprzyk (1997) indicated that the ease or difficulty of behavioral performance will affect a person’s behavioral intention. Guided by the TPB and based on the discussion above, the hypothesized model of this research is presented in Figure 1.

## Materials and Methods

### Data and Sample

This study used data from a larger study on people’s online behavior. The current study focused on HFS. The sample comprised 971 Chinese-speaking respondents in Hong Kong. In the sample, there were 473 (48.7%) male and 492 (50.7%) female respondents. There were 6 respondents (0.6%) who did not provide their gender and they were marked as missing values. The age of the respondents ranged from 14 to 34 years, mainly (93.2%) in the range of 19 to 24 years, and 26 (2.7%) respondents did not provide their age, with the mean age of 21.11 years.

### Instruments

The survey questionnaire for the current study was administered in Chinese. It comprised five scales, i.e., Social Justice, Vigilantism, HFS Intention, Positive Attitude toward HFS, and Fair (described in detail below). The scales of Social Justice, Vigilantism, Positive Attitude toward HFS, and Fair all comprised five Likert-type response options, namely, “Strongly Disagree,” “Disagree,” “Neutral,” “Agree,” and “Strongly Agree,” which were coded as 1,2,3,4, and 5, respectively. The scale of HFS Intention comprised five Likert-type response options of “Strongly Unwilling to,” “Unwilling to,” “Neutral,” “Willing to,” and “Strongly Willing to,” which were also coded as 1,2,3,4, and 5, respectively. For each question, respondents were instructed to “Please choose one answer and tick where appropriate.”

Respondents also indicated their daily online time with nine categories: “Never,” “Less Than 1 H,” “1–3 H,” “3–6 H,” “6–9 H,” “9–12 H,” “12–15 H,” “15–20 H,” and “More Than 20 H,” which were coded as 0 h, 0.5 h, 1.5 h, 4.5 h, 7.5 h, 10.5 h, 13.5 h, 17.5 h, and 22 h, respectively. Both gender and daily time spent online were used as control variables in this research.

#### Dependent Variable: Human Flesh Searching Intention

Participants were asked their likelihood to contribute to certain HFS activities (“items”). We adopted the items created by Chang and Leung (2015) after reviewing the HFS cases in the greater China region in 2003–2012. The twelve items were:

(1) Corruption activities among government officials;
(2) misconduct of government officials’ family members;
(3) sex scandals of government officials;
(4) minor crime issues;
(5) immoral activities;
(6) finding missing people;
(7) helping others to save life;
(8) sex scandals of artists;
(9) incidents about business activities;
(10) expression of personal negative emotions;
(11) helping police to solve certain crimes, and
(12) news about celebrities.

Most of the situations were crime or deviant related scenarios. The Cronbach’s Alpha was found in the current study to be 0.934.

#### Independent Variables

##### Attitude toward social justice

Five items were used to test participants’ attitudes toward social justice. These items were adopted from the Social Justice Scale developed by Torres-Harding et al. (2012) and included:

(1) It is important to make sure that all individuals and groups have a chance to speak and be heard, especially those from traditionally ignored, or marginalized groups;
(2) it is important to talk to others about societal systems of power, privilege, and oppression;
(3) it is important to try to change larger social conditions that cause individual suffering and impede well-being;
(4) it is important to help individuals and groups to pursue their chosen goals in life;
(5) it is important to support community organizations and institutions that help individuals and groups achieve their aims.

The reliability was also tested, and the Cronbach’s Alpha was 0.847.

##### Vigilantism

Participants were asked about their attitude toward vigilantism. Seven questions relating to vigilantism were selected from the confidence of criminal justice systems scales developed by Haas (2010). Participants were asked to answer whether they agree or disagree with statements below:

(1) People who kill armed robbers should not be blamed;
(2) it is sometimes ok for people to take justice into their own hands if they feel the police are unable to protect them;
(3) communities should organize themselves against criminals even if the police disagree with that;
(4) if the government is not successful in their fight against crime, citizens are justified to take the law into their own hands;
(5) citizens should take the law into their own hands more frequently;
(6) it is pointless to hand over a suspected criminal to the police because they will not bring the offender to justice, and
(7) I feel that taking the law into my own hands is justified by circumstances.

The Cronbach’s Alpha was 0.860.

##### Fairness

There were seventeen items used to evaluate participants’ attitude toward the fairness of the criminal justice system. Again, they were retrieved from the confidence of criminal justice systems scales developed by Haas (2010). Participants were asked whether they agree to seventeen statements relating to judges and the police:

(1) Judges treat people fairly;
(2) judges are trustworthy;
(3) I can count on the judges to take decisions that are best for society;
(4) I respect judges;
(5) judges deserve respect among citizens;
(6) if a judge passes a light sentence, he will have a good reason for that;
(7) judges’ verdicts are well deliberated;
(8) judges do their job well;
(9) judges know what is going on in society;
(10) the police are trustworthy;
(11) the police care about the well-being of every citizen;
(12) I can count on the police to take decisions that are best for society;
(13) the police take citizens seriously;
(14) if the police decide not to arrest someone, they will have a good reason;
(15) the police do their job well;
(16) the police are effective in combating crime, and
(17) the police are there when I need them.

We conducted a two-factor (judges and police) model for the Fairness scale and found that the correlation between these two factors is.55. Also, in the one-factor model, the item loadings were more than 0.5. As a result, the one-factor model was employed in this study. The Cronbach’s Alpha was 0.926.

##### Attitude toward human flesh searching

Six items were used in this research to test participants’ positive attitude toward HFS. Chang and Leung (2015) developed the original scale after they reviewed all the literature related to HFS in the Greater China region in 2003–2012. The six items were (1) HFS can maintain justice; (2) HFS can reveal the truth; (3) HFS can punish the bad guys; (4) HFS is very important; (5) HFS can compensate for the inadequacy of the current legal system and, (6) HFS serves justice by neglecting the influence of social hierarchy. The Cronbach’s Alpha was 0.850.

### Procedure

The data was collected using a face-to-face survey. The survey questionnaire was designed by the research team and was administered in Chinese. The questionnaire interviewers were trained before they started collecting the data. University students in Hong Kong were invited to participate in this survey (see section “Data and Sample”). The survey was conducted one to one or in a small group at university public spaces, mainly at the student canteen. Students participated in this research voluntarily and using their private time. Before the survey started, participants were provided an information sheet describing the project, the interview process, advantages and disadvantages of taking part in the research, information on de-identifying of the data and how the data will be used. The project was approved by the Human Ethical Review Committee at the City University of Hong Kong.

The measurement model was conducted using the multidimensional Graded Response Model (Samejima, 1997) with Mplus (Version 7.2) and the responses to items measuring the five latent variables were specified as ordered categorical. To test the hypothesized model, a two-step analysis was conducted. In the first step, the measurement model was conducted for the five latent variables with daily online time and gender as covariates using Mplus (Version 7.2); meanwhile, 50 sets of plausible values for each latent variable were generated. There were 19 (2.0%) cases with missing values for daily online time or gender. These data were excluded when generating plausible values. The Bayesian estimation approach was adopted for the above mentioned two analyses. In the second step, a path analysis was conducted using these 50 sets of plausible values, as well as the observed values of online time and gender, using Mplus (Version 7.2). By using plausible values, the measurement error was taken into consideration. The standard analysis for plausible value was conducted automatically using Mplus, with the parameter estimates averaged over 50 analyses. However, the indirect effect and total effect of the dependent variables were calculated using the command of “Model Constraint.” The following equations describe the hypothesized path model used in the current study:

$$\mathrm{HFS}\,\mathrm{Intention}=\beta_{10}+\beta_{11}\,\left(\mathrm{Positive}\,\mathrm{HFS}\,\mathrm{Attitude}\right)+\beta_{12}\,\left(\mathrm{Social}\,\mathrm{Justice}\right)+\beta_{13}\,\left(\text{Vigilantism}\right)+\beta_{14}\,\left(\text{Fair}\right)+$$

(1) $$\beta_{15}\,\left(\text{Gender}\right)+\beta_{16}\,\left(\mathrm{Daily}\,\mathrm{Online}\,\mathrm{Time}\right)+\varepsilon_{1}$$

(2) $$\mathrm{Positive}\,\mathrm{HFS}\,\mathrm{Attitude}=\beta_{20}+\beta_{21}\,\left(\mathrm{Social}\,\mathrm{Justice}\right)+\beta_{22}\,\left(\text{Vigilantism}\right)+\beta_{23}\,\left(\text{Fair}\right)+\beta_{24}\,\left(\text{Gender}\right)+\beta_{25}\,\left(\mathrm{Daily}\,\mathrm{Online}\,\mathrm{Time}\right)+\varepsilon_{2}$$

## Results

### Effect of Gender and Daily Online Time on HFS Intention and Attitude

A path analysis was conducted to test the hypothesized model using Mplus. The results showed that the hypothesized model was just identified [degree of freedom [d.f.] = 0] and no useful fit information was provided (Table 1). To release the degree of freedom, the non-significant effects were fixed at zero with the backward stepwise method based on the largest *P* values. According to the hypothesized Model (Model 1) result, the effect of gender on HFS Intention β_15_ was -0.002, with the largest *P* value of 0.949. Therefore, in Model 2 the β_15_ was fixed at zero. Likewise, β_25_, with the largest *P* value of 0.895 in Model 2, was fixed at zero in Model 3. By this analogy, all the non-significant coefficients were fixed at zero in Model 6, with β_23_ as the estimate with the largest *P* value of 0.044, which is significant at 0.05 level. The detailed information of the model constraint information is shown in Table 1. As is shown in the table, Model 6 was accompanied with the lowest AIC, BIC, and ABIC, which suggested that it was the best model. Also, the Chi-square tests for the change of Chi-square for adjacent models were all non-significant, which indicated later models cannot be rejected. Furthermore, the non-significant Chi-squared test of the model fit for Model 6 showed that the data fitted the model well. This result, on the other hand, showed that gender and daily online time had no significant effect on HFS Intention and HFS Positive Attitude.

**TABLE 1:** Model constraint information.

Model	AIC	BIC	ABIC	Chi-square (d.f., *P* value)	Chi-square change test (d.f., *P* value)	Estimate with largest *P* value (*P* Value)
Model 1	6783.588	6856.466	6808.827	0.000 (0, 1.000)	N/A	β_15_ = -0.002 (*P* = 0.949)
Model 2	6781.736	6849.756	6805.292	1.609 (1, 0.205)	N/A	β_25_ = 0.004 (*P* = 0.895)
Model 3	6779.977	6843.138	6801.851	0.860 (2, 0.651)	N/A*	B _24_ = -0.013 (*P* = 0.694)
Model 4	6778.385	6836.688	6798.576	0.917 (3, 0.821)	0.057 (1, 0.811)	B _12_ = 0.026 (*P* = 0.442)
Model 5	6777.834	6831.278	6796.342	1.585 (4, 0.812)	0.668 (1, 0.414)	B _16_ = 0.021 (*P* = 0.454)
Model 6	6776.564	6825.150	6793.391	2.145 (5, 0.829)	0.560 (1, 0.454)	β_23_ = -0.081 (*P* = 0.044)

Chi-square for the baseline model is 290.717 (d.f. = 11, *P* < 0.001). The estimates were standardized (STDYX). Model 1: Hypothesized model. Model 2: β_15_ = 0. Model 3: β_15_, β_25_ = 0. Model 4: β_15_, β_25_, and β_24_ = 0. Model 5: β_15_, β_25_, β_24_, and β_12_ = 0. Model 6: β_15_, β_25_, β_24_, β_12_, and β_16_ = 0. NA, Not Available. *The Chi-square value for Model 3 (d.f. = 2) was less than that for Model 2 (d.f. = 1), which suggested that Model 3 was better than Model 2.

### Effect of Social Justice, Vigilantism and Fair on HFS Intention and Attitude

**FIGURE 2:**
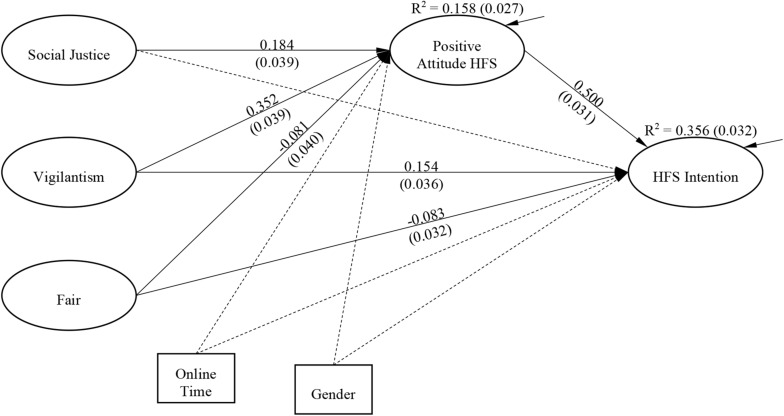
Final model [Fit Indices: Chi-Square = 2.145 (d.f. = 5, *p* = 0.829), RMSEA < 0.001, CFI > 0.96, TLI > 0.96, and SRMR < 0.01]. Note: 1. All the estimates were standardized (STDYX). The standard errors were presented in parentheses. 2. Two step analysis was conducted. The results of the path analysis shown above were obtained based on 50 sets of plausible value using Mplus. 3. The solid lines indicated significant effects at 0.05 level, and dashed lines indicated non-significant effects at 0.05 level and, therefore, were fixed at zero.

The result of the final path model is shown in Figure 2, and the total effect, direct, and indirect effect of Social Justice, Vigilantism and Fair on HFS Intention and Positive HFS Attitude are shown in Table 2. As is shown in the result, the effect of Positive Attitude toward HFS intention, among the concerned variables, was the strongest (standardized coefficient = 0.500). Vigilantism was also a strong predictor of HFS Intention. The total effect of Vigilantism to HFS Intention is 0.330, with direct effect as 0.154 and indirect effect via Positive HFS Attitude as 0.176. The effect of Fair to HFS Intention was found to be negative (total effect = -0.123, direct effect = -0.083, and indirect effect via Positive HFS Attitude = -0.040). However, no direct effect of Social Justice on HFS Intention was found. Social Justice exerted its effect via the Positive Attitude toward HFS, with a total effect (indirect) of 0.092.

**TABLE 2:** Total, direct and indirect effects of dependent variables.

Effect	Estimate	Standard error
Attitude to intention (total effect = direct effect)	0.500	0.031
Social justice to intention (total effect = indirect effect): Indirect effect via attitude	0.092	0.021
Vigilantism to intention (total effect):	0.330	0.036
Direct effect	0.154	0.036
Indirect effect via attitude	0.176	0.022
Fair to intention (total effect):	−0.123	0.036
Direct effect	−0.083	0.032
Indirect effect via attitude	−0.040	0.020
Social justice to attitude (total effect = direct effect)	0.184	0.039
Vigilantism to attitude (total effect = direct effect)	0.352	0.039
Fair to attitude (total effect = direct effect)	−0.081	0.040

*All the estimates are standardized (STDYX) and significant at 0.05 level. Attitude = Positive HFS Attitude. Intention = HFS Intention.*

Similarly, Vigilantism was the strongest predictor of Positive Attitude toward HFS (standardized coefficient = 0.354). Social Justice was also a positive predictor, with a standardized coefficient of 0.184. The effect of Fair to Positive HFS Attitude was negative (standardized coefficient = -0.081). In addition, the *R*-squared for Positive Attitude toward HFS was 0.158, and that for HFS Intention was 0.356.

## Discussion

From the results, we can argue that netizens who have an intention to contribute to HFS are those who have less confidence in the fairness of the criminal justice system and would take justice into their own hands, irrespective of gender and time spent accessing the internet. Similarly, for those who believe in social justice, if they are provided a tool that they think is efficient for them to realize justice, they also will tend to take justice into their own hands.

The results show that those who have less confidence in the criminal justice system are the ones with a higher intention to contribute to HFS and become netilantes. This is aligned with the result of existing research such as Chang and Poon (2017), Chia (2019b), and the concept model developed by Trottier (2019). These are the groups of people who do not have trust in judges and police and believe that people should take justice into their own hands if the legal system cannot protect them. While some of them might already be vigilantes in the real world, the internet provides netizens a new platform to realize the justice which they believe the criminal justice system will not be able to achieve. The intention will be reinforced if they have a positive attitude toward HFS and believe that HFS can help realize justice.

Aligned with the TPB, this research found that a positive attitude toward HFS is the strongest predictor of HFS intention. The HFS platform provides a space for netizens to speak out and contribute to their “justice.” Those who believe that the HFS platform provides them with a good way to maintain social justice, reveal truth, punish bad guys, and which can complement the inadequacy of the current legal system have a higher intention to conduct HFS. Indeed, as Gao (2016) argued, the internet has provided a platform for ordinary people to expose information that they were not able to do through traditional media. The HFS platform also provides a good medium for people to pursue their justice outside the traditional criminal justice system, especially for minor local cases that might not receive police attention.

The positive attitude toward HFS also works as a mediator. As mentioned earlier, it empowers those who do not have confidence in their current criminal justice system to take justice into their own hands online. For those who want to build their online reputation, they can publish their identity (real or fake) while disclosing crucial information. As some cases attract attention by traditional media (such as the 2013 Boston marathon bombing case and corruption cases in China), the netilante’s contribution to HFS will also be recognized online and possibly also in the media.

The beauty of the HFS platform is that netizens can choose to be identified or to remain anonymous by using a nickname or fake ID. The HFS platform provides those who do not want their real identity to appear on the platform, a channel to provide information. Netizens can hide behind the computer and not have to worry that they will be identified. This might explain why those who tend to have a higher attitude of social justice might not have an intention to contribute to HFS without the mediation of their positive attitude toward the HFS platform. That is, with confidence in the HFS, those who believe in social justice are empowered to contribute without worrying about being identified.

This research shows that for Chinese-speaking respondents in Hong Kong who want to contribute to “justice,” technology has provided them a good channel to do so. People, male and female, can take justice into their own hands using the HFS platform. It shows also that not all netizens are netilantes. HFS can be seen as a planned behavior by those netizens who see injustice and unfairness in society and/or who believe they can contribute to realize justice. The HFS platform gives them a good conduit to identify, investigate and even punish a suspect using their own means.

However, it is important that we be wary of the negative effect and ethical concerns that might come with HFS. Cases have already been reported of the wrong person targeted, causing serious damage to the reputation of the person and even leading to suicide (Chang et al., 2018). While HFS can fulfill the public’s right to know, it can only be regarded as legitimate when there is a balance between “the public’s right to know” and “the individual’s right to privacy” (Bu, 2008; Chang and Poon, 2017). As Chang and Poon (2017) argued, “over-justice” of netilantism can develop into a tyranny when the victim’s privacy is exploited in an incontrollable manner with no chance for self-defense.” As Zetter (2007) argues, activities in cyberspace are too hard to control once they have been initiated. Therefore, while netizens taking “justice” into their own hands might contribute to crime investigation, it is also important to have a second thought before contributing to such activities. There is a need for further studies into mitigation of the damage caused by netilantism. There is also a need for further research to establish whether people who conduct netilantism in western societies have similar characteristics and motivations as those identified in this study of participants in Hong Kong.

## Data Availability Statement

The raw data supporting the conclusions of this article will be made available by the authors, without undue reservation.

## Ethics Statement

The studies involving human participants were reviewed and approved by the Ethics Committee at the City University of Hong Kong. The patients/participants provided their written informed consent to participate in this study.

## Author Contributions

Both authors contributed to the article and approved the submitted version.

## Conflict of Interest

The authors declare that the research was conducted in the absence of any commercial or financial relationships that could be construed as a potential conflict of interest. The reviewer, KK, declared a shared affiliation, with no collaboration, with one of the authors, JZ, to the handling editor.
